# Vision-related quality of life is selectively affected by comorbidities in patients with geographic atrophy

**DOI:** 10.1186/s12886-023-02901-9

**Published:** 2023-04-12

**Authors:** Ditte-Marie Leegaard Holm, Marie Krogh Nielsen, Birte Bay Højsted, Torben Lykke Sørensen

**Affiliations:** 1 Clinical Eye Research Division, Department of Ophthalmology, Zealand University Hospital, Vestermarksvej 23, Roskilde, DK-4000 Denmark; 2 Faculty of Health and Medical Sciences, University of Copenhagen, Copenhagen, Denmark

**Keywords:** Age-related macular degeneration, Early treatment diabetic retinopathy study, Visual acuity, Vision-related quality of life, Visual function questionnaire 39, Comorbidities, Smoking

## Abstract

**Background:**

The atrophic late stage of age-related macular degeneration (AMD) is termed geographic atrophy (GA), and affects visual acuity (VA) as well as quality of life (QoL). Previous studies have found that best-corrected VA (BCVA), the standard vision assessment often underrepresents functional deficits. Therefore, the purpose of this study was to evaluate the correlation between atrophic lesion size, VA and QoL measured with the National Eye Institute Visual Function Questionnaire (VFQ-39) in a Danish population. Moreover, we wanted to evaluate the correlation between comorbidities, behavioural factors, and QoL.

**Methods:**

This was prospective clinical study of 51 patients with GA in one or both eyes, of these 45 patients had bilateral GA. Patients were consecutively included between April 2021 and February 2022. All patients filled in the VFQ-39 questionnaire except the subscales “ocular pain” and “peripheral vision.” Lesion size was measured from fundus autoflourescense images, and BCVA was assessed by the Early Treatment Diabetic Retinopathy Study (ETDRS) protocol.

**Results:**

We found an overall low score in each VFQ-39 subscale scores reflected by GA. Lesion size and VA were both significantly associated with all VFQ-39 subscale scores except for “general health.” VA showed a larger effect on QoL than lesion size. Chronic obstructive pulmonary disease (COPD) was associated with a lower score in the subscale score “general health” but none of the other subscale scores were affected. Cardiovascular disease (CVD) was associated with a lower BCVA as well as in QoL reflected in the subscale scores “poor general vision,” “near activities,” and “dependency” of VFQ-39.

**Conclusion:**

Both atrophic lesion size and visual acuity affects QoL in Danish patients with GA, who reports an overall poor QoL. CVD seems to have a negative effect on disease, as well as in VFQ-39 in several subscales, whereas COPD did not affect disease severity or vision-related subscales in VFQ-39.

## Background

Age-related macular degeneration (AMD) is a chronic progressive eye disorder involving the central retina, and it is a leading cause of vision impairment in the elderly [1, 2]. Geographic atrophy (GA) is a late stage of AMD, where progressive atrophy destroys the pigment epithelium, choriocapillaris and photoreceptors [1]. In the elderly over 70-years-old in Europe the prevalence of late AMD has been estimated to be 3.0% [3]. Moreover at the age of 85-years-old the incidence of GA is around 4 times higher compared to neovascular-AMD, and up to 22% of 90-years-old suffer from GA [4]. Currently, there is no treatment available to avoid or halt disease.

Geographic atrophy often occurs bilaterally, where atrophic lesions originates parafoveally from where the lesions can enlarge through the macular area and beyond. If GA is not involving fovea, best-corrected visual acuity (BCVA) may be rather good on a chart, but still affect daily functions such as reading, driving, cooking etc. due to delayed dark adaption, reduced contrast sensitivity and dense central scotomas. When the atrophic lesion involves the fovea, the patient will experience a further deterioration in visual function. Previous studies have assessed the association between visual acuity (VA), retinal extent of GA, and scores in visual function questionnaires (VFQ) in patients with GA [5–8]. Nevertheless, to our knowledge no studies have previously evaluated this in a Scandinavian population, and moreover no studies have previously investigated the association between VFQ, general health status reflected in existents of comorbidities nor smoking in a population with GA. Since most patients above the age of 70 have more than one chronic disease [9], our study provides important insights into how GA affects the vision-related quality of life in patients with comorbidities.

### Aims of the study

We wanted to evaluate what factors affects VFQ-39 in patients with GA. Firstly, we wanted to evaluate whether the size of GA or visual acuity had most impact on QoL in a Danish population. Secondly, we wanted to evaluate the correlation between smoking, presence of comorbidities, and VFQ-39-scores.

## Methods

### Study population

This was a prospective clinical study that took place at the Department of Ophthalmology, Zealand University Hospital, Denmark. All potential patients were explained the nature and purpose of the study, and all patients gave oral and written consent. The study followed the tenets of the Declaration of Helsinki and was approved by the Regional Ethics Committee (reference number: SJ-736). All patients were questioned about medical history, current medication, and tobacco use. In addition medical history as well as current and previous medication was available through the electronic patient chart. Patients completed a paper-based validated Danish translation of the National Eye Institute Visual Functioning Questionnaire 39 (NEI VFQ-39). Questions were allowed to be read out and interview guide was followed [10]. Ophthalmological examinations included BCVA, slit-lamp bio-microscopy, indirect dilated fundoscopy, spectral domain optical coherence tomography (OCT) and fundus autofluorescence image. Optical coherence tomography angiography (OCT-A) was performed in order to rule out neovascular AMD (Heidelberg Engineering, Heidelberg, Germany).

Patients were included between April 2021 and February 2022. We included patients with any geographic atrophy in either one or both eyes. Exclusions criteria were (1) sign of current or former neovascular AMD in any eye; (2) if the patients did not speak or understand Danish language; (3) vision less than 25 ETDRS letters.

### Visual acuity

BCVA was obtained with Early Treatment Diabetic Retinopathy Study (ETDRS) performed by certified vision examiner in accordance with protocols [11]. VA was measured one eye at a time, and in the analyses the eye with best visual acuity was study eye.

### Imaging and analysis

All the images with geographic atrophy were evaluated by a trained doctor using Region Finder software version 2.6.4.0 (Heidelberg Engineering, Heidelberg, Germany). The doctor manually identified a point within the GA (pixels seen as dark areas) and increased until threshold in order to include adjacent pixels, so the GA delineated from the normal areas. In cases of multifocal lesions, only lesions ≥ 0.05 mm^2^ were included in the total lesion size. If the geographic lesion were larger than 30° of the retina, all seen GA was measured. In order to delineate the geographic lesions from blood vessels and macula pigment, constraints were manually placed.

### Questionnaire

Visual Function Questionnaire (VFQ-39) is used worldwide and measures vision-related quality-of-life [12]. It contains 25 items and additionally 14 more items that are optional. The total score on each item ranges from 0 to 100 (where 100 represents best function). It contains 12 subscales concerning general health, general vision, ocular pain, near activities, distance activities, social functioning, mental health, role difficulties, dependency, driving, color vision, and peripheral vision (Table 1) [13]. The Danish version is validated for patients with AMD, except the questions regarding ocular pain and peripheral vision [10], and therefore questions regarding these were excluded from this study. The patients were allowed to have missing information on some questions, but one questionnaire was excluded from the analyses since 13 answers were left out.

**Table 1:** Summary of the subscales of VFQ-39

Subscale	Description
General health	Rating of overall health
General vision	Rating of binocular corrected vision
Near activities	Difficulties with reading, doing needlework, cooking, handiwork, finding certain things on a crowded shelf due to vision problems
Distance activities	Difficulties with reading traffic signs, going on stairs in dim light, going to the cinema or sports events due to vision problems
Social functioning	Difficulties with recognising facial expressions, going out to social events due to vision problems
Mental health	Worries about vision, frustrations about vision, loss of control due to vision problems, afraid of embarrassing oneself or others
Role difficulties	Limitations in duration of work due to vision problems, limitations in things wanting to due to vision problems
Dependency	Staying home due to vision problems, need a lot of help from others due to vision problems, not leaving home alone
Driving	Difficulties with driving in familiar places in daylight, difficulties driving at night, in bad weather, rush hour or on highway
Color vision	Difficulties finding or picking out matching clothes

## Data analysis

Descriptive statistics were calculated, and continuous data were presented as mean and standard deviation, categorical data is given as percentages. The total score for each VFQ-39 subscale was calculated, as well as composite score. For each score, we calculated the Spearmann’s correlation coefficient with both visual acuity in best-seeing eye, and with largest atrophic lesion size. One-sample T-test was performed comparing the VFQ-39 in each category to the presence or absence of comorbidities. Statistical significance was set at P < 0.05.

## Results

**Fig. 1:**
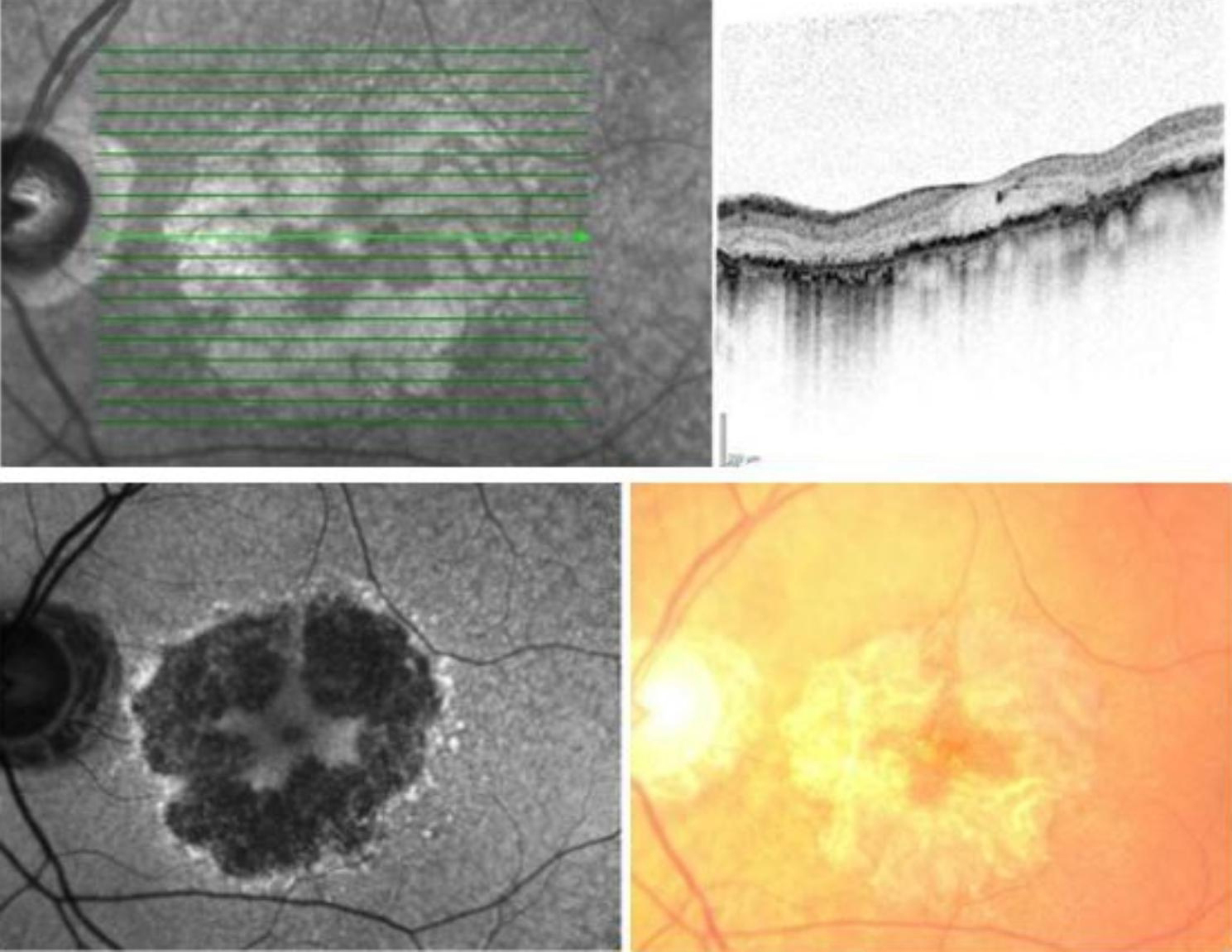
Illustrations of geographic atrophy. Images illustrates left eye of 78-year-old male with geographic atrophy. Infrared image (top left), optic coherence tomography (top right), autofluorescense image (bottom left) and fundus photo (bottom right)

The study consisted of data from 51 patients (Table 2), and 45 of the patients had bilateral GA. The mean age was 79.3 ± 7.1 years, and a total of 63% of the patients were female. A total of 51% were former smokers and 4.3% active smokers. In 23 of the 28 cases of active or former smokers, the patients provided information on amount and duration of smoking. The mean number of pack-years was 22.76 (Range 0.6–66). We found that 47% were suffering from hypertension, 35% from CVD, 16% of hypercholesterolemia, and 14% of chronic obstructive pulmonary disease (COPD). Only four patients (8%) had type 2 diabetes, and from these two suffered from hypertension. The best-corrected visual acuity letter score (ETDRS) in the best eye was 64.0 ± 18.4 letters, and smallest lesion of GA was 6.6 ± 7.9 mm^2^. Figure 1 illustrates geographic atrophy.

**Table 2:** Patients characteristics, N = 51

Age, mean (SD)		79.3 (7.1)
Gender, %	Female	63%
	Male	37%
Smoking, %	Never	45%
	Former	51%
	Active	4%
Hypertension, %		47%
Hypercholesterolemia, %		16%
Cardiovascular disease, %		35%
Chronic obstructive pulmonary disease, %		14%
Type 2 diabetes, %		8%
BMI, mean (SD)		26 (4)
VA in best seeing eye, mean (SD)		64.0 (18.4)
VA in worst seeing eye, mean (SD)		42.7 (21.3)
Smallest lesion area, mean (SD)		6.6 (7.9)
Largest lesion area, mean (SD)		9.1 (8.8)

SD: standard deviation, BMI: Body mass index, VA: visual acuity

The VFQ measures are shown in Table 3. One patient’s answers of the questionnaire were excluded due to only answering 38.2% of the questionnaire. When assessing the VFQ-39 the subscale scores that were lowest, were near activities (52.3), general vision (54.4), and mental health (55.8).

**Table 3:** Mean VFQ-39 scores

VFQ measures	Mean (SD)
Composite score	65.0 (18.1)
General Health	67.0 (19.7)
General Vision	54.4 (17.8)
Near Activities	52.3 (20.6)
Distance Activities	59.9 (23.7)
Social Function	77.5 (21.4)
Mental Health	55.8 (24.5)
Role Difficulties	60.9 (25.1)
Dependency	74.4 (25.6)
Color Vision	84.9 (21.2)

Mean of the subscale scores in VFQ-39 (National Eye Institute Visual Function Questionnaire) for patients with geographic atrophy in one or both eyes

**Fig. 2:**
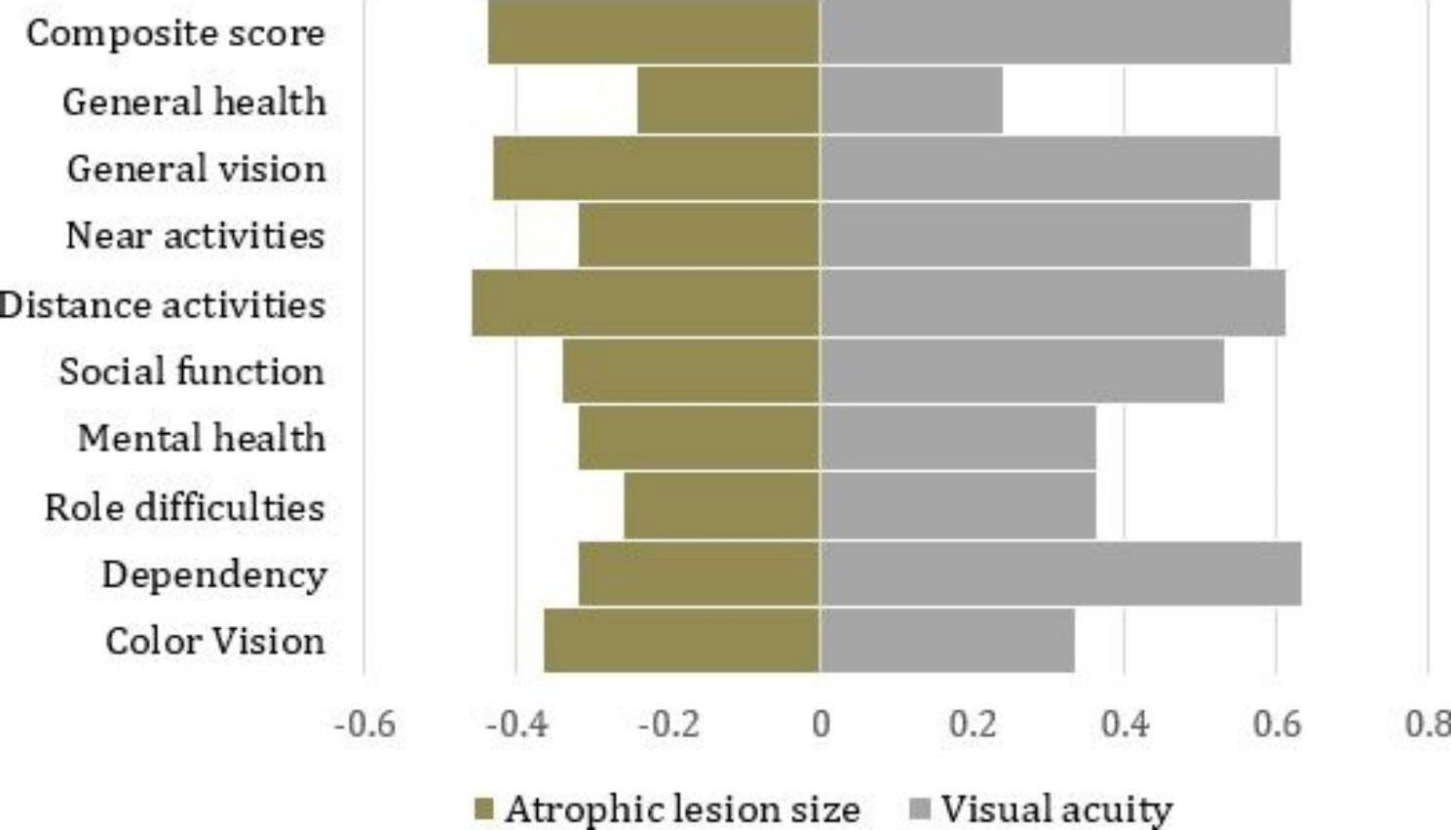
Association between vision-related quality of life and atrophic lesion size and visual acuity. Shows the correlation between the different subscale scores and atrophic lesion and visual acuity

Figure 2 shows the correlation between VFQ-39 and atrophic lesion size, and the correlation between VFQ-39 and BCVA. In our study, BCVA showed a larger effect on QoL than lesion size. When evaluating effect size, defined by a previous study [14], the correlation between “near activity” and BCVA showed a moderate effect size, and lesion size a small effect size, which was the same for “dependency” and “social function.”

### Comorbidities and smoking

**Fig. 3:**
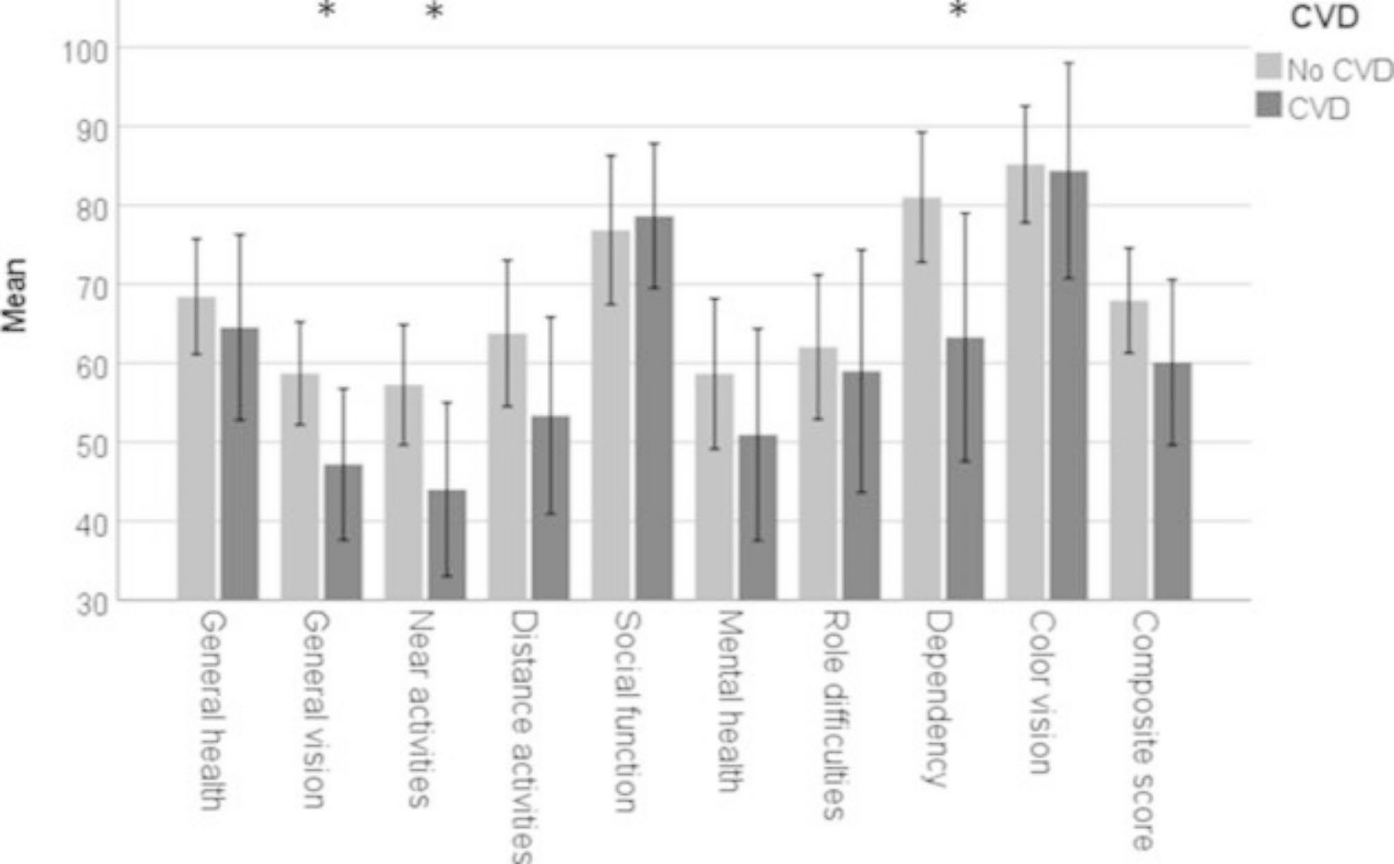
Association between vision-related quality of life and cardiovascular disease. Shows the difference in mean and confidence intervals of each subscale scores for patients with and without CVD. Significant differences is marked with asterix. VFQ-39 (the National Eye Institute Visual Function Questionnaire), CVD (cardiovascular disease)

**Fig. 4:**
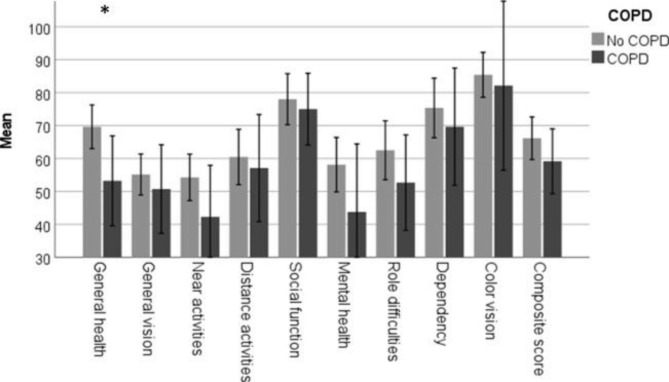
Association between vision-related quality of life and chronic obstructive pulmonary disease. Shows the difference in mean and confidence intervals of the subscale scores for patients with and without COPD. Significant differences is marked with asterix. VFQ-39 (the National Eye Institute Visual Function Questionnaire), COPD (chronic obstructive pulmonary disease)

**Fig. 5:**
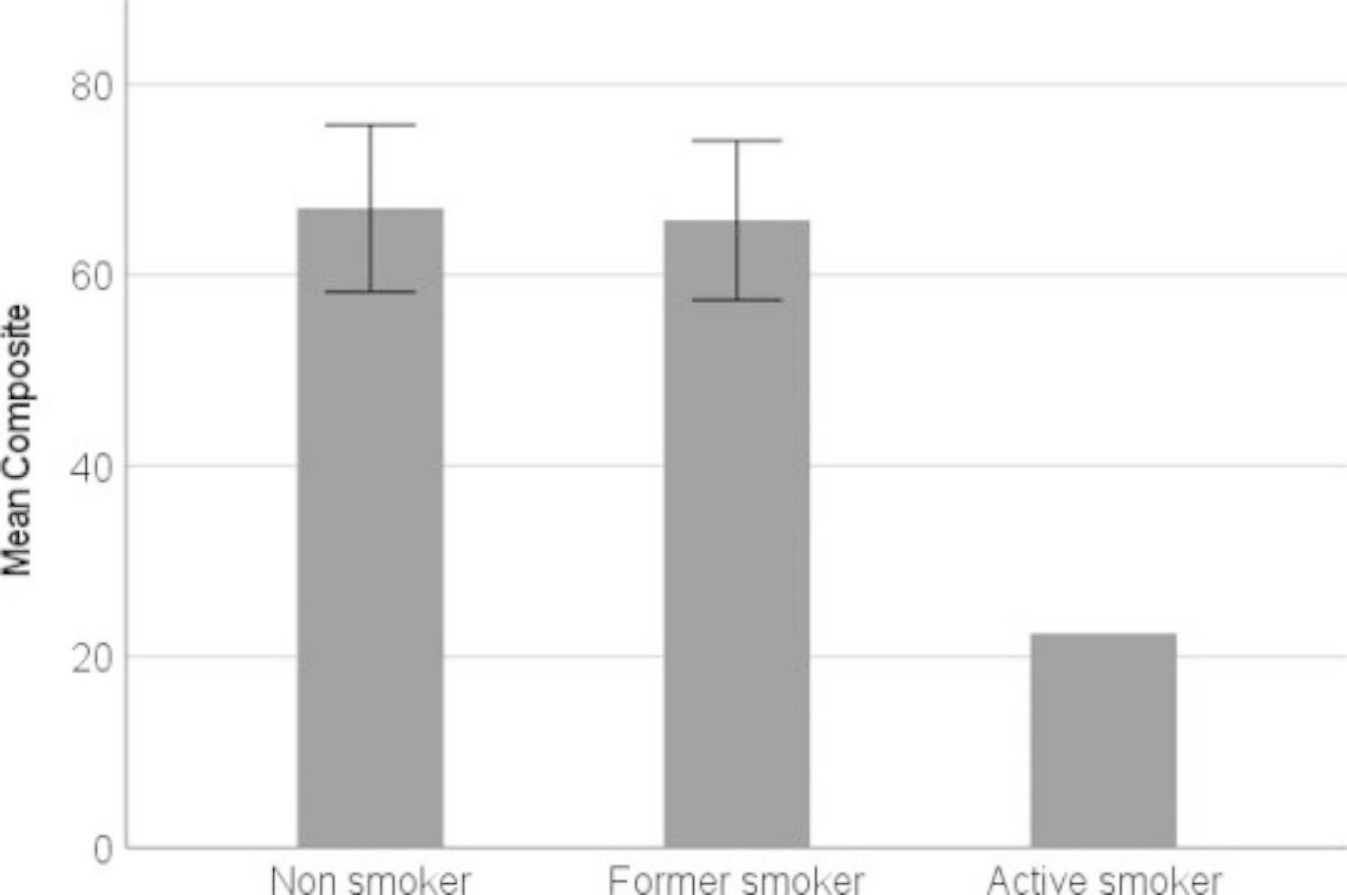
Association between mean composite score of the VFQ-39 and smoking status. Due to small numbers, it was not possible to establish 95% for ”Active smoker.” VFQ-39 (the National Eye Institute Visual Function Questionnaire)

The presence of CVD was associated with poor BCVA (mean difference = 11.2 letters, 95% CI:0.8–21.7, p = 0.036). Patients with CVD had lower scores concerning “general vision,” “near activities,” and “dependency” compared to patients without CVD (Fig. 3). COPD was associated with poor “general health,” but not with other subscale scores (Fig. 4), and there was no difference in BCVA for patients with and without COPD (mean difference = 4.2 95% CI:-11.0-19.3, p = 0.055). Active smoking was associated with worse composite score compared to non or previous smoking, however the number of active smokers were small (n = 2). The result is presented in Fig. 5.

## Discussion

We found that Danish patients with geographic atrophy (GA) have a low vision-related quality of life and that BCVA had a slightly stronger correlation to QoL compared to atrophic lesion size. Furthermore we found that CVD was associated with poor BCVA, “general vision,” “near activities,” and “dependency.” The presence of COPD was associated with poor “general health” but not with other subscale scores of the VFQ-39. This might indicate that different comorbidities can influence disease severity and self-perceived visual quality of life.

In our study, the mean composite score of VFQ was 65.0, which is comparable with composite scores of 61.7-69.96 estimated by previous studies, respectively [5, 6, 15]. In a previous study including patients with few if any drusen, their results on the individual subscale scores were considerably higher compared to ours (e.g. “General vision”: 83, “Near activities”: 93, “Dependency”: 99). Our results hereby indicate that GA is reflected in VFQ-39 compared to patients with sparse AMD changes.

When addressing the associations of either BCVA and VFQ or GA and VFQ a German study also found the strongest association between BCVA and the composite score of VFQ-25 [6]. In the Mahalo study, weak correlations were found for both BCVA and GA. However, in contrast to the present study in the Mahalo study, the study eye had the poorest visual function [5], which makes comparisons with our study difficult. Unlike our results a Spanish study estimated the mean composite score to be 46.67 and found a stronger association with lesion size [7]. BVCA in our study was 64 (≈ 0.4 logmar value), which is comparable with the German study that estimated the BVCA to be 0.30 for the eye with the best visual function (logmar value). In our study, the mean of BCVA for the eye with the worst visual function was 42.7, which is also comparable with the Mahalo study [5] that estimated the BCVA to be 48.0. When patients are suffering from irreversible vision loss, vision rehabilitation can be useful and help to preserve daily functions of the individual affected. It is likely that the possibility to receive such rehabilitation affects vision-related quality of life in Denmark, where the government provides such services when VA for the eye with the best visual function drops below 60 ETDRS letters. It remains unknown how many of the patients in our study have had vision rehabilitation, but vision rehabilitation does not seem to have influenced our results, since our results are comparable with foreign studies that do not necessarily have vision rehabilitation.

We found that CVD was associated with poor BCVA, and when evaluating the associations between the different subscale scores of VFQ-39 and CVD, patients with CVD had lower scores of “general vision,” “near activities” and “general health” compared to patients without CVD. To our knowledge, no other studies have previously investigated the association between comorbidities, BCVA, and atrophic lesion size. A previous study that did not restrict on type of AMD (30.7% with dry AMD) found that the composite score of VFQ-25 was associated with hypertension but not with CVD, which they also investigated for [8]. We did not find any association between the composite score and CVD either, but between certain subscale scores and CVD, and perhaps we did so due to poor BCVA in patients with CVD.

We found that COPD was only associated with a low score in “general health” in the VFQ-39 but not with the other subscale scores, and that may be explained by the fact that patients with COPD are reported to suffer from comorbidities and are frequently physically inactive [16]. Interestingly, a previous study found that QoL in patients with AMD was lower compared to patients with COPD [17], and thereby it is understandable that our patients report poor “general health,” if they are suffering from both GA and COPD.

Smoking is a very well known risk factor for AMD [18, 19], but the association between smoking and VFQ-39 in patients with GA has to our knowledge not previously been studied. In our study active smoking was associated with a lower mean composite score compared to non or previous smoking. A previous study did not find any association between smoking and the composite score of VFQ-25 [8]. However, unlike our study, they did not restrict on type of AMD, and it is unknown whether they stratified in previous and active smoking, moreover it cannot be ruled out that we observed an association between current smoking and VFQ-39 due to small numbers. Considering that both previous and active smoking is a risk factor for AMD [19], it is interesting that there was no statistical difference in the mean of composite scores between non- or previous smoking.

Limitations in our study include the observational character of the study and hereby we can only speculate of causality. Since this was a clinical study our patients may not represent the general population with GA, and thereby selection bias cannot be ruled out.

In conclusion, we found that both atrophic lesion size and visual acuity affect the quality of life in Danish patients with GA, who reports an overall poor QoL reflected in VFQ-39. The presence of CVD seems to have a negative effect on disease, as well as in several subscale scores of the VFQ-39, whereas COPD did not affect disease severity or vision-related subscales in VFQ-39, but was only reflected in poor “general health.”

## Data Availability

The datasets used during the current study are available from the corresponding author on reasonable request.

## List of Abbreviations

AMD: Age-related macular degeneration
GA: Geographic atrophy
BMI: Body Mass Index
QoL: Quality of life
VA: Visual acuity
COPD: Chronic obstructive lung disease
VFQ-39: Visual Function Questionnaire-39
OCT: Optical coherence tomography
OCT-A: Optical coherence tomography angiography
ETDRS: Early Treatment Diabetic Retinopathy Study
BCVA: Best-corrected visual acuity
CVD: Cardiovascular disease

## References

[1] Lim LS (2012). Age-related macular degeneration. Lancet.

[2] Mitchell P (2018). Age-related macular degeneration. Lancet.

[3] Colijn JM (2017). Prevalence of age-related Macular Degeneration in Europe: the past and the future. Ophthalmology.

[4] Sacconi R (2017). A review of current and future management of Geographic Atrophy. Ophthalmol Ther.

[5] Sivaprasad S (2018). Reliability and construct validity of the NEI VFQ-25 in a subset of patients with Geographic Atrophy from the phase 2 Mahalo Study. Am J Ophthalmol.

[6] Künzel SH (2020). Determinants of Quality of Life in Geographic Atrophy secondary to age-related Macular Degeneration. Invest Ophthalmol Vis Sci.

[7] Burguera-Giménez N (2020). Multimodal evaluation of visual function in Geographic Atrophy versus normal eyes. Clin Ophthalmol.

[8] Chatziralli I (2017). Risk factors for poor quality of life among patients with age-related Macular Degeneration. Semin Ophthalmol.

[9] Barnett K (2012). Epidemiology of multimorbidity and implications for health care, research, and medical education: a cross-sectional study. Lancet.

[10] Sørensen MS (2011). Danish version of visual function Questionnaire-25 and its use in age-related macular degeneration. Dan Med Bull.

[11] Kaiser PK. *Prospective evaluation of visual acuity assessment: a comparison of snellen versus ETDRS charts in clinical practice (An AOS Thesis)* Trans Am Ophthalmol Soc, 2009. 107: p. 311 – 24. PMC2814576 20126505

[12] Mangione CM (2001). Development of the 25-item National Eye Institute visual function questionnaire. Arch Ophthalmol.

[13] Version. 2000. The National Eye Institute 25-Item Visual Function Questionnaire (VFQ-25) Available from: https://www.rand.org/content/dam/rand/www/external/health/surveys_tools/vfq/vfq25_manual.pdf.

[14] Sullivan GM, Feinn R (2012). Using effect size-or why the P value is not enough. J Grad Med Educ.

[15] Heier JS (2020). Visual function decline resulting from Geographic Atrophy: results from the Chroma and Spectri Phase 3 trials. Ophthalmol Retina.

[16] Decramer M, Janssens W, Miravitlles M (2012). Chronic obstructive pulmonary disease. Lancet.

[17] Williams RA (1998). The psychosocial impact of macular degeneration. Arch Ophthalmol.

[18] Chakravarthy U (2010). Clinical risk factors for age-related macular degeneration: a systematic review and meta-analysis. BMC Ophthalmol.

[19] Velilla S et al. *Smoking and age-related macular degeneration: review and update* J Ophthalmol, 2013. 2013: p. 895147. 10.1155/2013/895147 PMC3866712 24368940

